# Application research on YOLOv5 model based on Lightweight Atrous Attention Module in brain tumor MRI image segmentation

**DOI:** 10.3389/fmed.2025.1660445

**Published:** 2025-10-09

**Authors:** Tao Yang, Jinghui Chen, Lianxin Xie, Lanlan Yang, Chengbin Ye, Hongjia Zhao

**Affiliations:** ^1^The First Clinical Medical College, The Affiliated People's Hospital of Fujian University of Traditional Chinese Medicine, Fuzhou, Fujian, China; ^2^Department of Medical Imaging, The Affiliated People's Hospital of Fujian University of Traditional Chinese Medicine, Fuzhou, Fujian, China; ^3^The Affiliated People's Hospital of Fujian University of Traditional Chinese Medicine, Fuzhou, Fujian, China

**Keywords:** artificial intelligence, image segmentation, brain tumor, magnetic resonanceimaging (MRI), YOLOv5S, Lightweight Atrous Attention Module (LAAM)

## Abstract

**Objective:**

To enhance the segmentation accuracy and computational efficiency of brain tumor magnetic resonance imaging (MRI) images, this study proposes a novel Lightweight Atrous Attention Module (LAAM) that integrates the Convolutional Block Attention Module (CBAM) with an Atrous Spatial Pyramid Pooling (ASPP) structure. The LAAM was integrated into the YOLOv5s model to enhance its performance, aiming to boost accuracy and recall while keeping computational efficiency.

**Methods:**

This study utilized two publicly available meningioma and glioma MRI datasets from Kaggle. The LAAM incorporates depthwise separable convolutions, dual attention mechanisms, and residual connections to reduce computational complexity while enhancing feature extraction capabilities. The modified YOLOv5s model was trained and validated via five-fold cross-validation, with performance comparisons conducted against the original YOLOv5s architecture and other optimized models.

**Results:**

The enhanced YOLOv5s-LAAM model demonstrated superior performance, achieving a precision of 92.3 %, a recall rate of 90.4 %, and an mAP@50 score of 0.925. Concurrently, the model exhibited significantly reduced computational demands, with the GFLOPs reduced by 15 % compared to the original YOLOv5s-ASPP baseline.

**Conclusion:**

The integration of the LAAM significantly enhances the YOLOv5s model's segmentation capabilities for brain tumor MRI images, making it a valuable tool for clinical diagnosis and treatment planning. The lightweight design ensures effective deployment in resource-constrained environments while maintaining high computational performance.

## 1 Introduction

Brain tumors, as malignant diseases posing severe threats to human health, continue to drive advancements in medical research due to their high mortality rates (1). Epidemiological studies indicate that although brain tumors constitute only 1.5 % of all malignant tumor cases, their mortality rate reaches 3 % (2). Magnetic resonance imaging (MRI), with its superior soft tissue resolution compared to computed tomography (CT), has become a core technology for tumor detection (3, 4). This technology can accurately distinguish tumors from normal tissues, providing critical evidence for tumor classification, treatment planning, and prognosis evaluation (5, 6).

Although conventional imaging techniques can initially screen for tumors, they still fall short in critical aspects such as precisely defining invasive margins, analyzing tumor heterogeneity, and evaluating treatment responses. While manual segmentation can be used for routine diagnosis, its low efficiency and subjective nature make it difficult to meet the high standards of precision medicine (7, 8). Current clinical practice predominantly relies on manual tumor delineation, which remains a laborious and time-consuming process (9, 10).

With continuous advancements in artificial intelligence (AI), AI-driven medical image analysis technology is revolutionizing diagnostic paradigms (11). Deep learning algorithms and computer vision have automated tumor region segmentation. This has greatly improved image analysis efficiency and established a standardized diagnostic process with less human bias. Studies have shown that CNN and U-net segmentation models can efficiently segment brain tumors in clinical settings (12).

In summary, tumor segmentation studies using CNN and U-Net have achieved remarkable results but are still plagued by model complexity and high computational costs. Given the clinical demand for real-time segmentation, there is an urgent need for more efficient and accurate algorithms. The YOLO framework, introduced in 2016 as a one-stage object detection method, leverages a single neural network to achieve end-to-end detection and directly predict target positions, demonstrating significant advantages in generalization ability and efficiency (13).

In our previous study, we preliminarily explored the application of the YOLOv5s model for brain tumor segmentation in MRI images. By integrating the Atrous Spatial Pyramid Pooling (ASPP) module and the Convolutional Block Attention Module (CBAM), we optimized the YOLOv5s architecture, resulting in a significant improvement in segmentation accuracy. The ASPP module is adept at capturing multi-scale contextual information from images. Yet, its reliance on standard convolution operations leads to a hefty parameter count and high computational complexity. When dealing with high-resolution medical images, the ASPP module's computational demands soar, which in turn slows down the model's inference speed, making it hard to satisfy clinical real-time requirements. Furthermore, the ASPP's method of fusing features from different scales is somewhat simplistic, potentially neglecting the synergistic optimization between local details and global features (14, 15).

The Convolutional Block Attention Module, which combines channel attention (CAM) and spatial attention (SAM) mechanisms, dynamically boosts the weights of key features. This enhances the model's ability to focus on tumor regions (16). However, CBAM has limitations in segmenting complex backgrounds or small targets. Its channel attention mechanism may weaken the expression of local feature differences, and the spatial attention mechanism is susceptible to interference from artifacts or low-contrast area (17, 18). Furthermore, CBAM's sequential computational structure introduces additional computational latency, thereby restricting its applicability in lightweight deployment scenarios (19, 20).

Building on prior studies, we focus on designing a novel module, the Lightweight Atrous Attention Module (LAAM). It aims to combine the strengths of ASPP and CBAM while overcoming their limitations.

Despite YOLO evolving to the 11th generation, YOLOv5 stands out with its lightweight architecture, flexible module design, and efficient deployment. Its compact size and low computational load enable fast inference on general-purpose GPUs and edge devices, meeting clinical diagnosis real-time requirements. Moreover, YOLO5's highly modular structure offers significant flexibility for integrating custom components like attention mechanisms or feature fusion modules, driving continuous algorithm iteration and optimization.

Compared to YOLOv5, The commonly used YOLOv8 and YOLOv11 are more complex in model structure and parameters. This boosts their performance in general detection tasks but also raises computational costs, slows inference, and increases hardware demands, making rapid medical deployment difficult (21, 22). Given the limited medical image data and high annotation costs, complex models are more prone to overfitting, which harms generalization. Additionally, their deep networks and complex connections reduce model interpretability and flexibility, hindering fine-tuning for specific tasks.

Therefore, we choose YOLOv5 as the foundation to leverage its lightweight, scalable, and easily deployable advantages. This aligns with our goal of model lightweight optimization and helps highlight the proposed module's effectiveness. By integrating the LAAM, which combines ASPP and CBAM advantages into YOLOv5s, we aim to enhance the model's ability to extract and recognize brain tumor features without significantly increasing computational burden, better meeting clinical needs.

The principal contributions of this study can be summarized as follows:

(1) We propose a novel The Lightweight Atrous Attention Module (LAAM) that synergistically integrates multi-scale atrous convolutions (ASPP) with enhanced attention mechanisms, achieving both efficient feature extraction and dynamic weight adjustment. The LAAM architecture employs depthwise separable convolutions and residual connections to significantly reduce computational complexity, while enhancing the expressive capacity of critical features through streamlined channel-spatial attention mechanisms. Compared to conventional ASPP and CBAM, LAAM achieves significant recall rate improvement while maintaining high precision, effectively addressing the critical challenge of balancing computational efficiency with segmentation performance in medical image analysis.
(2) The LAAM replaces the original Spatial Pyramid Pooling (SPP) structure, integrating multi-scale contextual information with lightweight design principles. Its lightweight ASPP sub-module employs depthwise separable convolutions with varying dilation rates to reduce parameter count, while the enhanced CBAM sub-module streamlines attention computations through bottleneck structures and 1 × 1 convolutions, effectively minimizing computational load. Experimental results demonstrate that this design maintains high recall rates and robustness even under the stringent Intersection over Union (IoU) = 0.9 threshold, outperforming conventional ASPP models while demonstrating suitability for resource-constrained clinical environments.

The remainder of this paper is organized as follows: **Section 2** reviews the strengths and limitations of the ASPP and CBAM, along with the innovative improvements proposed in this study. **Section 3** details the model training methodology and evaluation metrics. **Section 4** presents the experimental results and facilitates in-depth analysis. Finally, **Section 5** discusses the experimental findings, draws conclusions, and outlines future research directions.

## 2 Improved methodology

### 2.1 Module analysis

In our previous study (23), we individually integrated ASPP, Coordinate Attention (CA), and CBAM into the YOLOv5 architecture. Experimental results demonstrated that the model embedding only the ASPP achieved optimal segmentation performance at an IoU threshold of 0.6, with a precision of 93.7 %, recall rate of 89.1 %, and mAP@50 score of 92.9 %. In medical image segmentation tasks, high recall rates ensure comprehensive detection of tumor regions while minimizing missed diagnoses. Even with high precision, low recall rates may lead to partial tumor region omission. However, improving precision remains equally critical, necessitating further research to enhance recall performance. Ablation experiments from previous studies demonstrated the effectiveness of ASPP and CBAM in boosting model capabilities. Therefore, this study focuses on analyzing the ASPP and CBAM to address these dual objectives.

The ASPP and CBAM demonstrate significant advantages in feature extraction and attention mechanisms, yet exhibit certain limitations. The ASPP captures contextual information across varying receptive fields through multi-scale atrous convolutions, effectively enhancing the model's adaptability to multi-scale targets (24). Kumar (25) conducted ablation experiments integrating their novel ASPP with the baseline Tiny YOLOv7 model, achieving significant performance gains of 17 % improvement in precision, 1 % increase in recall rate, 11 % enhancement in F1-score, and 12.55 % boost in mean Average Precision (mAP) compared to the baseline, demonstrating multiplicative improvements in detection capability. However, this approach incurs high computational complexity, with substantial increases in parameter count and computational load, particularly with high-resolution inputs or large channel dimensions, potentially compromising training and inference efficiency. Furthermore, the ASPP exhibits limited capacity in capturing localized detail information, as its design prioritizes the extraction of global contextual features. This may lead to the neglect of certain critical local characteristics, potentially creating performance bottlenecks in tasks requiring fine-grained localization.

The CBAM dynamically adjusts feature weights through its channel and spatial attention mechanisms, effectively enhancing critical features while suppressing redundant information. Chen (26) conducted ablation experiments integrating CBAM with the baseline YOLOv8n model, achieving a 0.3 % improvement in mAP@50–95 compared to the original baseline. However, the computational process of its attention mechanism is relatively complex, particularly the 7 × 7 convolution operation in spatial attention, which significantly increases computational overhead and may adversely affect the model's real-time performance. Furthermore, the CBAM demonstrates limited capability in capturing multi-scale information, as it primarily relies on local feature dependencies and inter-channel relationships. This design may lead to suboptimal performance in large-scale scene understanding tasks, particularly in scenarios requiring global contextual information, where its effectiveness might underperform compared to the ASPP.

### 2.2 Innovative improvement strategies

In this study, we propose further enhancements to the ASPP and CBAM by adopting two distinct improvement strategies. The specific approaches are outlined as follows:

(1) Atrous Pyramid Convolution with Attention Module (APCM): by integrating the strengths of ASPP and CBAM, this module achieves dual enhancement of multi-scale feature extraction and attention mechanisms. The ASPP component employs 1 × 1 convolutions, dilated convolutions with varying dilation rates, and global pooling to capture multi-scale information from input features, thus effectively enhancing the model's adaptability to target size variations. The CBAM dynamically adjusts feature weights through channel and spatial attention mechanisms, enhancing critical features while suppressing redundant information. By concatenating the outputs of CBAM and ASPP followed by 1 × 1 convolutional fusion, the proposed APCM preserves multi-scale feature extraction capabilities while refining features via attention mechanisms. However, the increased computational complexity may compromise inference speed.
(2) Lightweight Atrous Attention Module (LAAM): this module enhances feature representation through efficient multi-scale convolutions and attention mechanisms while maintaining low computational complexity. Its core design comprises a lightweight ASPP branch and an enhanced CBAM branch. The former employs depthwise separable convolutions to capture local context, while the latter utilizes multi-scale dilated convolutions for broader receptive fields. Additionally, simplified channel and spatial attention mechanisms dynamically adjust feature weights, amplifying critical features and suppressing redundant information. Furthermore, LAAM incorporates a residual connection that employs a 1 × 1 convolution to integrate the input features with the attention-refined features. This design preserves original feature information, mitigates gradient vanishing, and enhances training stability.

### 2.3 Module integration strategy

**Figure 1:**
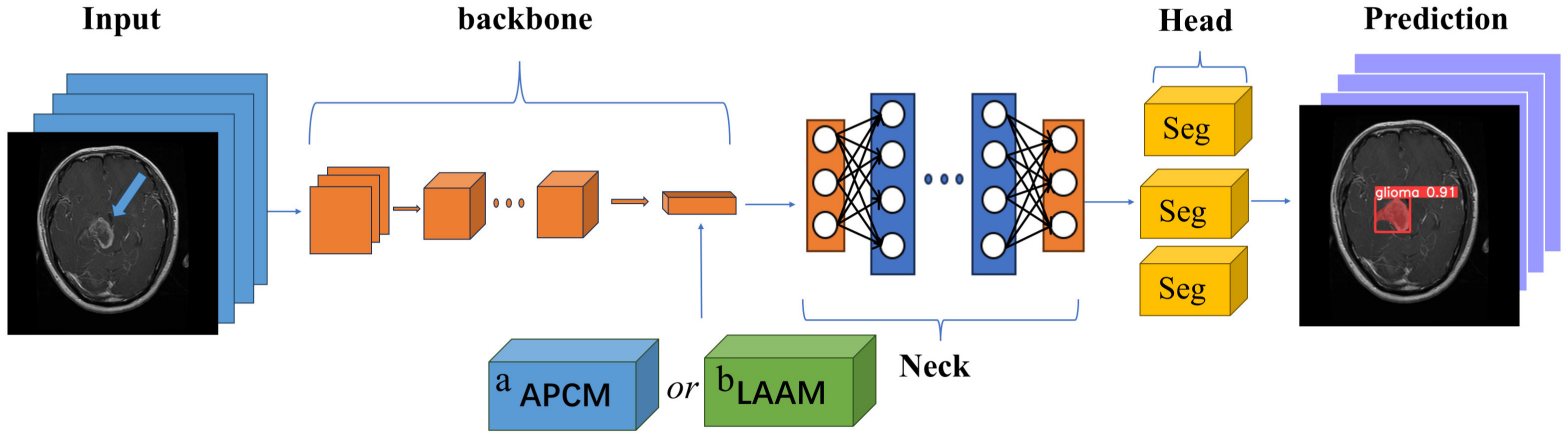
Module placement diagram. a APCM module; b LAAM module. The SPP structure in the backbone of the original model is replaced by the APCM or LAAM module. (a) APCM module: It combines standard ASPP multi-scale convolution with the CBAM attention mechanism, and uses residual connections to enhance feature representation. (b) LAAM module: it employs a lightweight ASPP (depthwise separable convolution) and a simplified CBAM structure. This approach reduces computation while maintaining multi-scale perception and attention weight optimization.

Figure 1 illustrates the module integration strategy of this study. Both the APCM and LAAM modules are used to replace the SPP structure at the end of the Backbone network in the original YOLOv5s model. The downsampled high-level feature maps extracted at the end of the Backbone are fed into the APCM or LAAM modules. These modules enhance the features at this critical point. The output is then passed to the Neck for feature fusion and finally to the Head for prediction. This integration method aims to embed enhancement modules at the key feature extraction point of YOLOv5s without altering its original architecture, thereby achieving precise performance improvement.

The SPP module employs fixed-size pooling operations to capture multi-scale features. However, its limited capability in capturing local details and inability to dynamically adjust feature weights (27, 28) restrict its performance in scenarios requiring precise localization. In contrast, the APCM module integrates multi-scale dilated convolutions (ASPP) to capture contextual information across varying receptive fields, combined with CBAM's channel and spatial attention mechanisms to dynamically enhance salient features while suppressing redundant information. This design not only effectively addresses target size variations and complex background interference, but also expands the receptive field while maintaining feature map resolution. Furthermore, the APCM preserves original feature information through residual connections, thereby mitigating the gradient vanishing problem in deep networks.

Compared to the SPP module, the LAAM achieves efficient multi-scale feature extraction and attention mechanism integration through a lightweight ASPP branch and an enhanced CBAM branch. The lightweight ASPP branch employs depthwise separable convolutions to significantly reduce parameter count and computational complexity, while employing dilated convolutions with varying dilation rates to capture multi-scale contextual information. The enhanced CBAM branch dynamically adjusts feature weights through streamlined channel and spatial attention mechanisms, amplifying critical features while suppressing redundant information. Furthermore, by incorporating residual connections, the LAAM preserves the original feature information, mitigates the vanishing gradient problem, and enhances training stability of the model.

To investigate the performance impact of the two modified modules on the model, ablation experiments were designed, with the specific experimental protocol outlined in Table 1. To validate the necessity of residual connections, we conduct ablation studies by comparing Lightweight Atrous Pyramid Convolution with Multi-scale Attention (LAPCM) and Lightweight Local-Attention Atrous Module (LLAAM) with their residual-equipped counterparts (APCM and LAAM).

**Table 1:** Design of ablation experiments.

**Experiment name**	**Module composition**	**Design objective**
YOLOv5s	Original YOLOv5s	Provide baseline performance
APCM	Standard ASPP + Standard CBAM + Residual Connections	Benchmark complete APCM module as performance upper bound
LAPCM	Standard ASPP + Standard CBAM	Validate the necessity of residual connections in APCM
LASPP	Lightweight ASPP	Test basic performance of lightweight ASPP
LLAAM	Lightweight ASPP + Enhanced CBAM	Verify compatibility between lightweight ASPP and enhanced CBAM
LAAM	Lightweight ASPP + Enhanced CBAM + Residual Connections	Evaluate final effectiveness of complete lightweight LAAM module
ASPP	Standard ASPP	Compare performance gap between lightweight ASPP and standard ASPP

### 2.4 Atrous pyramid convolution with attention module

The Atrous Pyramid Convolution with Attention Module (APCM) is designed to enhance feature representation in complex scenes by integrating multi-scale feature extraction with attention mechanisms. Its core architecture combines the advantages of multi-scale atrous convolutions (ASPP) and channel-spatial attention mechanisms (CBAM), while residual connections are introduced to strengthen information retention and propagation. Specifically, the APCM captures multi-scale contextual information from input features through its ASPP branch utilizing atrous convolutions with varying dilation rates, thereby effectively addressing challenges posed by target scale variations and complex background interference. The ASPP branch consists of four parallel sub-modules, whose outputs are fused via concatenation followed by a 1 × 1 convolution to generate integrated multi-scale feature representations. This design significantly enhances the model's adaptability to multi-scale targets.

**Figure 2:**
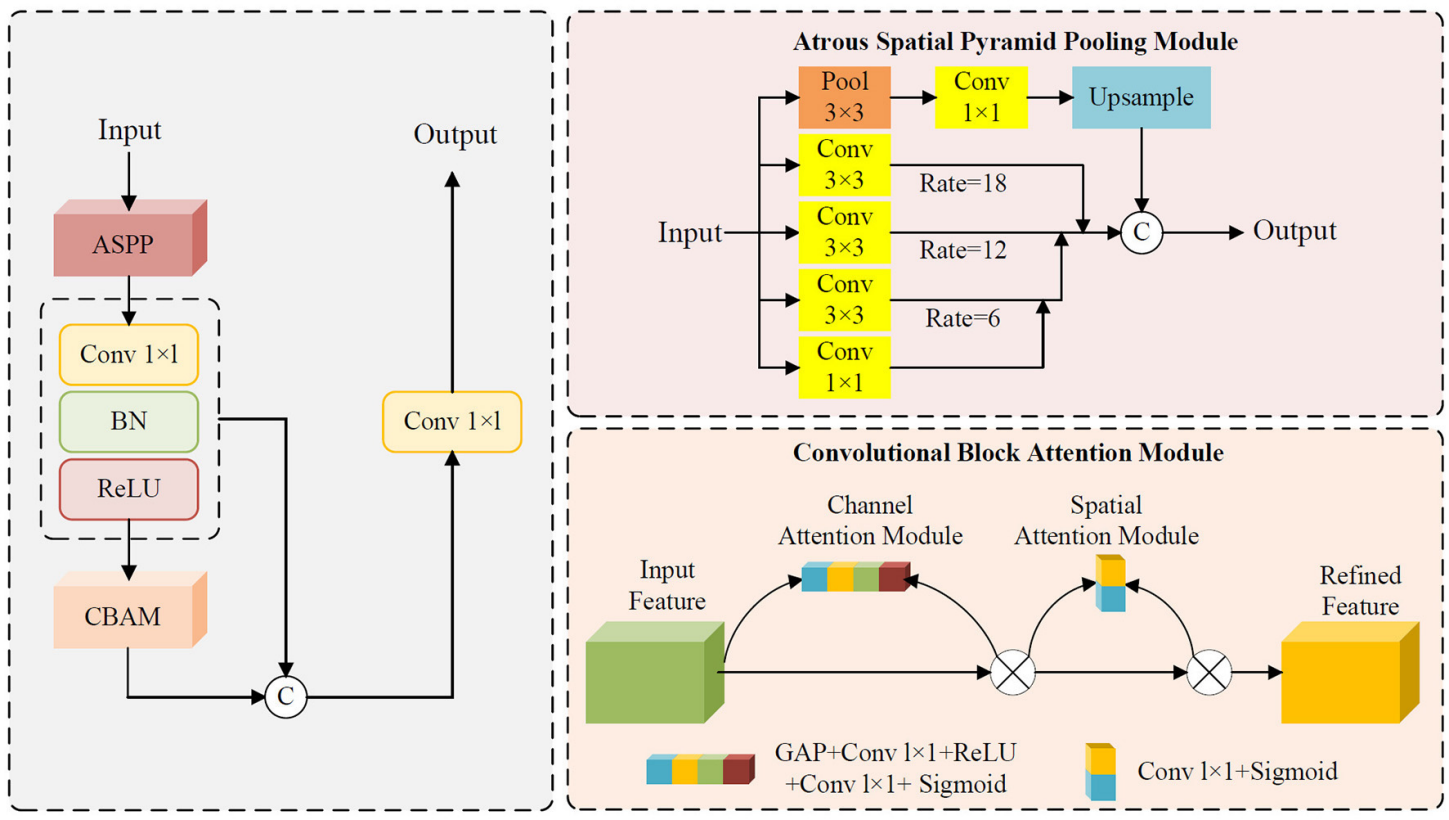
Schematic structure of APCM.

Building upon the ASPP branch, the APCM further incorporates a CBAM branch to dynamically adjust feature weights through channel-wise and spatial attention mechanisms. The CAM computes channel importance using GAP followed by fully connected layers, while the spatial attention derives spatial significance by aggregating mean and max values along the channel dimension. The combined attention mask is multiplied with the ASPP output features, thereby further amplifying critical features and suppressing redundant information. Furthermore, the APCM employs residual connections to sum the input features with the attention-enhanced features. This design not only preserves the original feature information but also mitigates the vanishing gradient problem in deep networks, improving both training stability and generalization capability of the model. The schematic structure of APCM is illustrated in Figure 2. The pseudocode of the APCM module is provided in Appendix as Algorithm A1.

### 2.5 Lightweight atrous attention module

**Figure 3:**
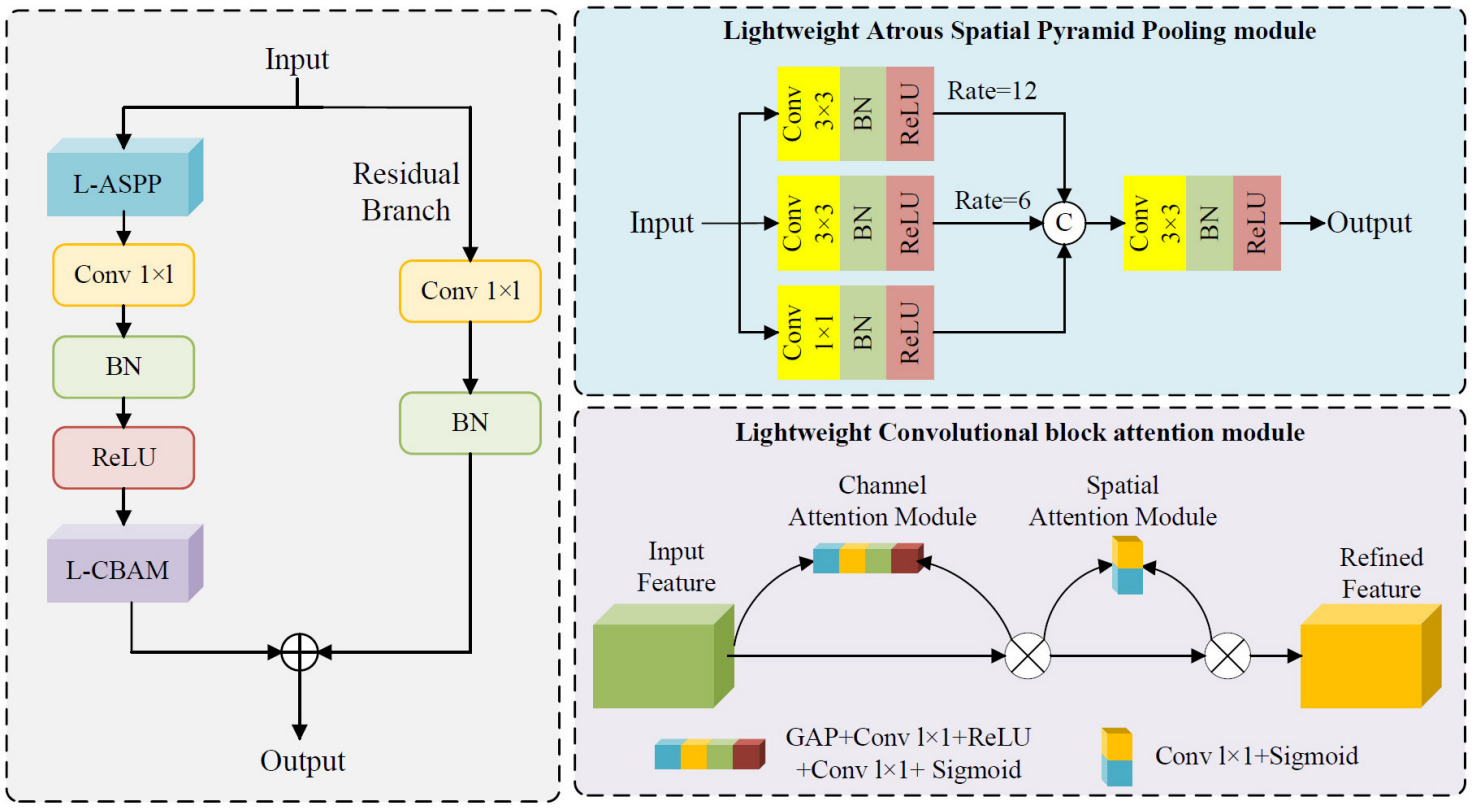
Schematic structure of LAAM.

The Lightweight Atrous Attention Module (LAAM) is designed to enhance feature representation in complex scenes by integrating multi-scale feature extraction with attention mechanisms, while maintaining low computational complexity. Its core design comprises a lightweight ASPP branch, an enhanced CBAM branch, and residual connections, carefully balancing computational efficiency and feature discriminability. The ASPP branch in LAAM employs depthwise separable convolutions for multi-scale feature extraction. Specifically, it captures context at varying ranges through 1 × 1 convolutions, 3 × 3 convolutions with dilation rates of 6 and 12, significantly reducing parameter count and computational overhead while preserving multi-scale feature diversity. The enhanced CBAM branch dynamically adjusts feature weights through streamlined channel and spatial attention mechanisms. The channel attention computes channel-wise importance via GAP combined with a bottleneck structure, while the spatial attention generates a spatial weight map using 1 × 1 convolutions. This design further reduces computational complexity while strengthening the representational capacity of critical features. Additionally, the LAAM incorporates residual connections where input features are adjusted to match the channel dimensions of the output features through 1 × 1 convolutions and batch normalization. These adjusted features are then summed with the attention-enhanced outputs, preserving original feature information and mitigating the vanishing gradient problem, thereby improving the model's training stability and generalization capability. The schematic structure of LAAM is depicted in Figure 3. The pseudocode of the LAAM module is provided in Appendix as Algorithm A2.

### 2.6 Comparative analysis between APCM module and LAAM module

The APCM and LAAM modules both aim to enhance feature representation by integrating multi-scale atrous convolution and attention mechanisms, but they differ significantly in design, structure, and information processing.

The APCM module, performance-oriented, uses a sequential coupling process. It leverages standard-convolution-based ASPP branches for multi-scale context extraction, passes features to the CBAM for attention modulation, and fuses them with original features via residual connections, ensuring complete feature modulation but with high computational complexity.

In contrast, the LAAM module focuses on lightweight and efficient design. Its highly structured and streamlined process uses depthwise separable convolution in ASPP branches to reduce parameters and computational load. It also simplifies the attention mechanism for compact computation, integrates it with lightweight ASPP branches, and minimizes redundant calculations. This allows LAAM to maintain multi-scale semantic capture while achieving a more efficient and compact information-processing workflow, catering to resource-constrained scenarios.

## 3 Materials and methods

### 3.1 Overall technology roadmap

**Figure 4:**
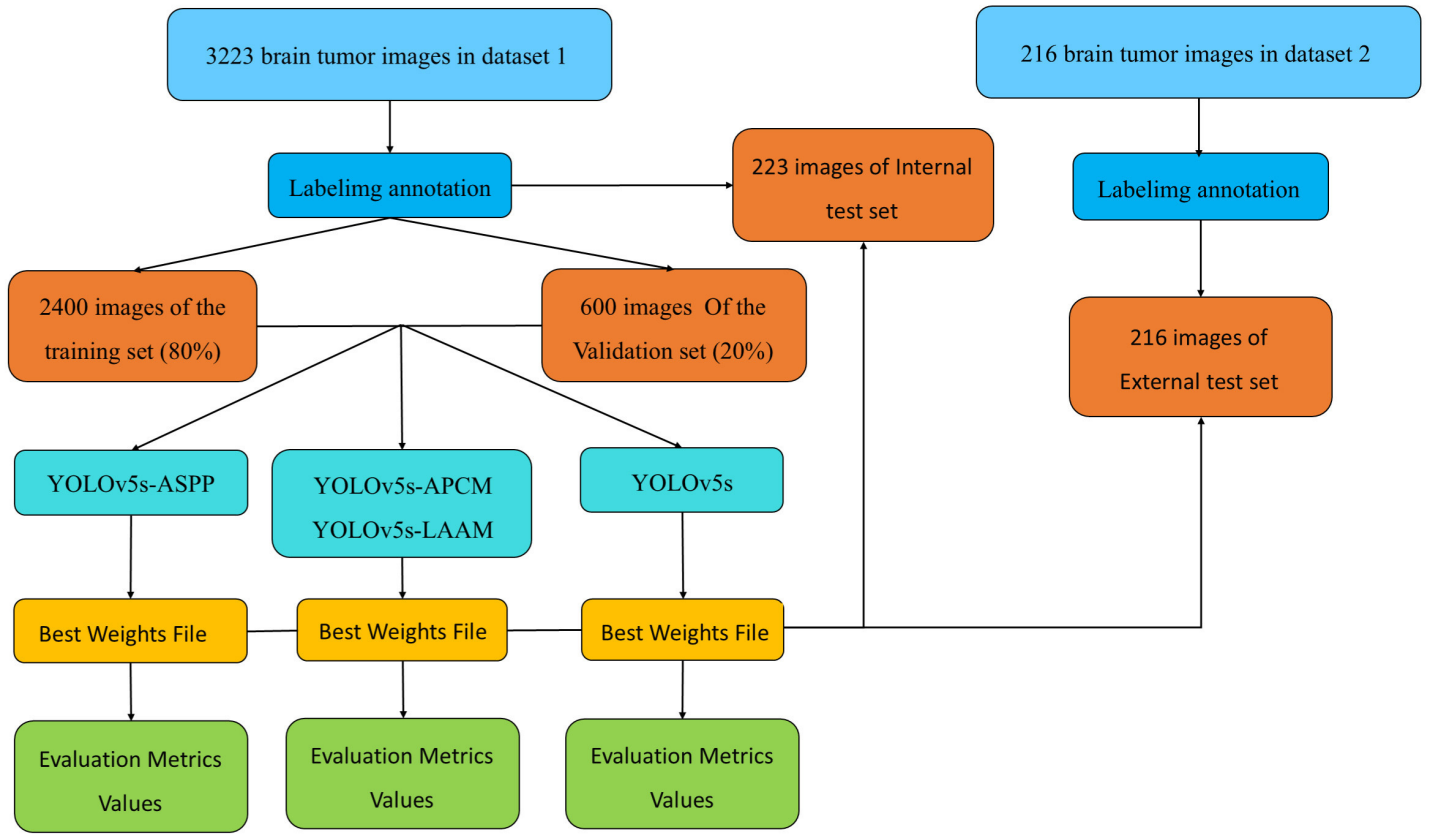
Technical roadmap of this study.

The overall technical route of this study is shown in Figure 4 and consists of four main stages: data preparation, model construction, training with internal validation, and testing with internal and external datasets. First, we obtained two brain tumor MRI image datasets from public sources and standardized them. Then, from Dataset 1, we randomly selected 3,000 images, which were divided into training and validation sets in a ratio of 8:2. The remaining 233 images were used as the internal test set for model training, optimization, and preliminary performance evaluation. Dataset 2 served as the external test set. Based on the YOLOv5s framework, we constructed baseline and multiple improved model variants, trained using five-fold cross-validation to obtain robust best weight files. Finally, we conducted a comprehensive performance evaluation of the best models using the unseen internal test set and the independent external test set to fully assess their generalization ability.

### 3.2 Data acquisition

The medical imaging data used in this study are derived from two public brain tumor MRI datasets on the Kaggle platform. The first dataset (29), referred to as Dataset 1 in this study, comprises a total of 3,223 brain tumor MRI images, including 1,581 gliomas and 1,642 meningiomas. It is utilized for model training and for testing model performance using an internal test set. The second dataset (30), containing 101 gliomas and 115 meningiomas, serves as an external test set for evaluating model performance.

In this study, we conducted necessary preprocessing on the brain tumor MRI images input into the YOLOv5s model for the segmentation task. The preprocessing mainly includes size standardization and pixel normalization. Firstly, the original images of different sizes are uniformly scaled to 640 × 640 pixels to meet the network input requirements. Subsequently, the pixel values are linearly normalized from the range of 0–255 to the interval [0, 1] to accelerate convergence and enhance training stability.

All images were pre-annotated for segmentation targets by two radiologists with the title of attending physician or above using Labellmg. The annotation results were verified on-site by two radiologists with the title of associate chief physician or above to ensure experimental accuracy.

**Figure 5:**
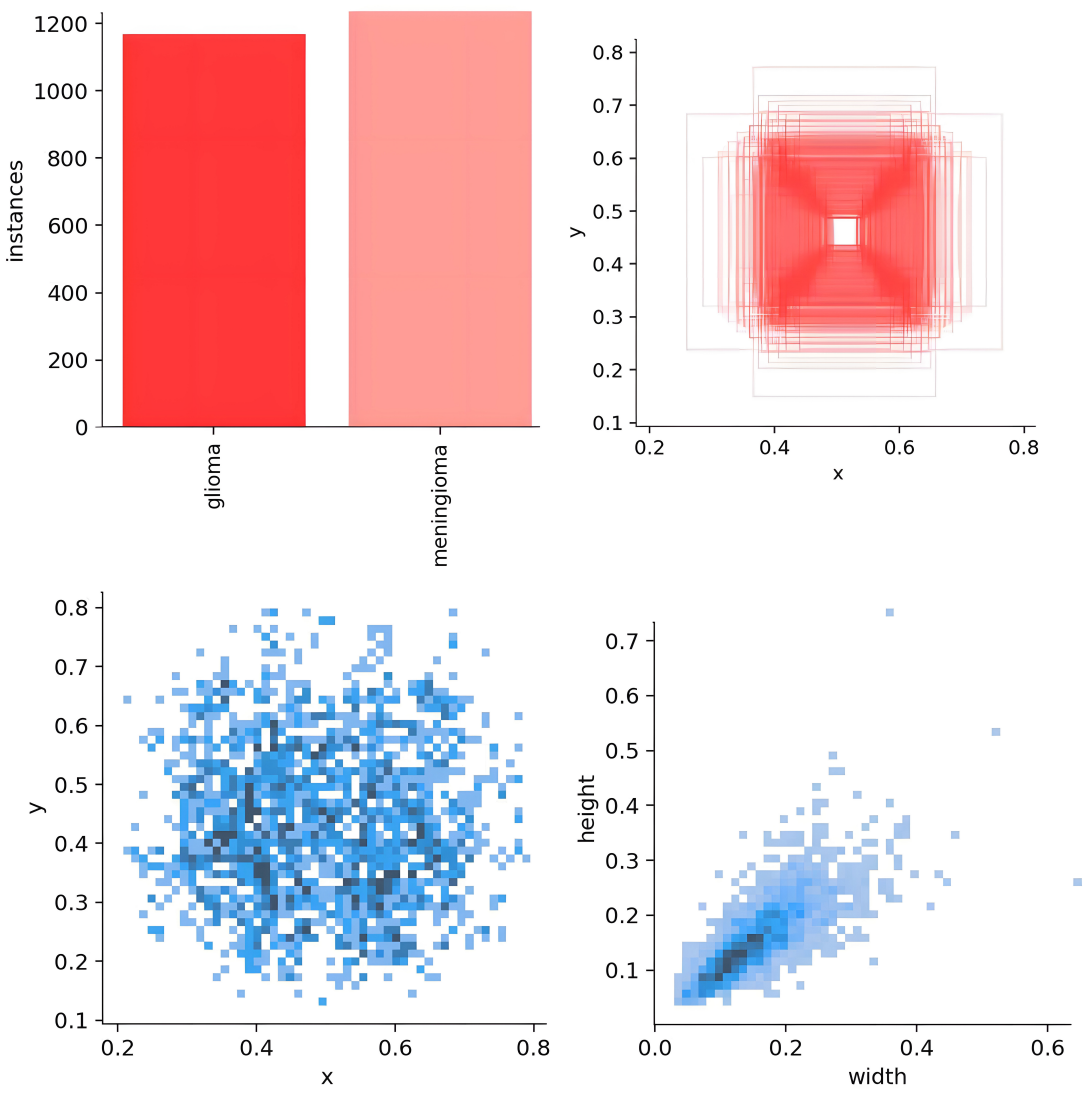
Analysis of bounding box distribution and dataset characteristics.

**Figure 6:**
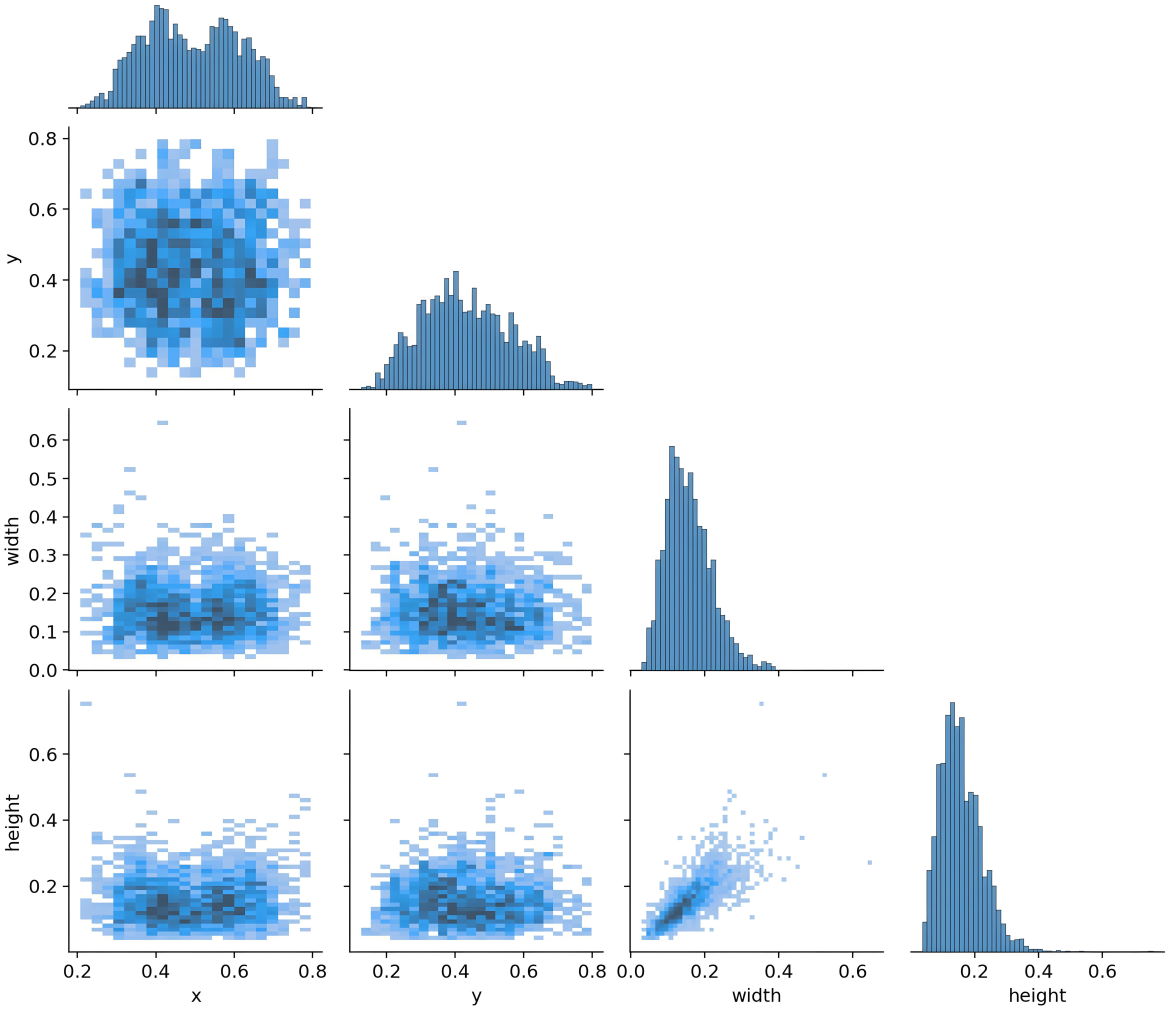
Target correlation graph in the dataset.

For model training, we implemented a five-fold cross-validation strategy. From Dataset 1, 3,000 images were randomly selected and equally divided into five subsets (600 images each). Each subset was further partitioned into training and validation sets at an 8:2 ratio. During each validation fold, four subsets were combined as the training set, while the remaining subset served as the validation set. The remaining 223 independent test images were reserved for evaluating the segmentation performance of the optimal model. The distribution characteristics of annotations in the training dataset and the inter-class correlation analysis results are presented in Figures 5, 6, respectively.

As shown in Figure 5, the scatter plot presents the key distribution characteristics of the annotation boxes. Using Matplotlib, the axes were automatically scaled to the actual distribution range of the data to reveal the details more clearly. The specific explanations are as follows: (1) distribution of the target center point (left sub-figure): *x*-axis 0.2–0.8, *y*-axis 0.2–0.6, indicating that the tumor center is concentrated in the center of the MRI image, without edge targets. This is in line with the characteristics of clinical scans, as the head is always positioned at the center point of the magnetic field. (2) Distribution of the target width and height (right sub-figure): axes 0.2–0.6, showing that the tumor size is medium and uniform, without extreme outliers; the clustering of points indicates that the aspect ratio is nearly symmetrical, which is consistent with the typical morphology of gliomas and meningiomas.

Figure 6 shows the correlation diagram of the labels of the training dataset. Each sub-figure presents the correlation of the normalized bounding box parameters (*x, y*, width, height). The *x*-axis range of all sub-figures is consistent with the bottom row, and the *y*-axis range is consistent with the left column, ensuring the consistency of the overall comparison. The histograms on the diagonal represent the distribution of individual variables. The *x*-axis corresponds to the range of the value of that variable, for example, width is 0.0–0.6, and the *y*-axis represents the frequency. In the histogram, the *y*-axis represents the frequency of target boxes in each interval, but no specific scale values are shown. The purpose is to emphasize the distribution trend rather than the absolute quantity.

### 3.3 Experimental parameters

All experiments were conducted on a computing system equipped with a 13th Gen Intel^®^ Core™ i7-13620H processor (2.40 GHz), an NVIDIA GeForce RTX 4060 Laptop GPU, and 16.0 GB RAM. The YOLOv5s and YOLOv8s architectures were implemented using PyTorch (Python 3.8.7, PyTorch 2.0.0, and CUDA 11.8).

Seven model variants (YOLOv5s, YOLOv5s-ASPP, YOLOv5s-LASPP, YOLOv5s-LAPCM, YOLOv5s-APCM, YOLOv5s-LLAAM, and YOLOv5s-LAAM) were trained across five datasets with distinct data distributions using stochastic gradient descent optimization. The training protocol employed a batch size of 8, initial learning rate of 1 × 10^−2^, 100 training epochs, and an IoU threshold of 0.6.

### 3.4 Experimental evaluation index

Drawing on previous studies (31), in this study, the model performance was measured using Precision, Recall, mAP@50 and GFLOPs. These evaluation metrics, similar to the Dice Similarity Coefficient (DSC), respectively reflect different aspects of the model performance. They are complementary to each other. DSC focuses on the consistency of pixel-level segmentation results with the true annotations in terms of spatial overlap, and is suitable for evaluating the quality of fine segmentation based on pixel-wise classification. In target detection and instance segmentation tasks, mAP@50 is a more general comprehensive evaluation metric. This metric calculates the area under the precision-recall curve at a specific IoU threshold to comprehensively assess the overall performance of localization accuracy and classification confidence. Therefore, mAP@50 can not only reflect localization accuracy but also reliability of recognition, and is a more comprehensive assessment of the overall performance of detection and segmentation.

Precision is defined as the proportion of samples predicted as positive (True Positive, TP) among all samples predicted as positive. It measures the extent to which the model's positive predictions are truly correct. A high Precision indicates a low rate of False Positives. Recall, on the other hand, is the proportion of actual positive samples (True Positive) that are correctly identified by the model. It emphasizes the model's ability to detect all true positive lesions. A high Recall indicates a low rate of False Negatives. In segmentation tasks, P represents the proportion of predicted pixels that are correctly classified as the target, while R represents the proportion of actual target pixels that are correctly predicted as the target. The specific calculation formulas are shown in (1) and (2).


(1) $$\begin{array}{l} \text{Precision}=\frac{TP}{TP+FP} \end{array}$$

(2) $$\begin{array}{l} \text{Recall}=\frac{TP}{TP+FN} \end{array}$$

mAP@50 represents the average value of the recall rate (True Positive Rate, TPR) when the precision reaches 50 %. It measures the proportion of predicted results from the model that have an Intersection over Union (IoU) greater than or equal to 0.5 while localizing the targets and assigning confidence scores.

A high mAP@50 indicates that the model can accurately identify targets with minimal deviation in position prediction in object detection tasks. It is an important criterion for evaluating model performance, especially in real-time applications where precision and efficiency are of great concern.

GFLOPs is a commonly used metric for measuring the computational load of deep learning models. A higher GFLOPs value for a model implies that it requires more computational resources and potentially more powerful hardware to support real-time or near-real-time inference.

After identifying the candidate models with excellent performance in the evaluation metrics through ablation experiments, this study further assesses their efficiency in actual deployment environments by comparing and analyzing the inference speed of the final selected models using the following three key time indicators:

Pre-process time: the time taken to process and convert raw input data to meet the model input requirements. This usually involves image scaling, normalization, channel conversion, and data loading onto the computing device.
Inference time: the core indicator of model inference speed, referring to the time the model spends on forward propagation computations of the pre-processed input tensor to generate raw output results. This directly reflects the model's computational complexity and architectural efficiency.
NMS time: the time required to decode and filter the model's raw prediction results, mainly through non-maximum suppression operations. This process eliminates redundant predictions and retains the most credible detection results, making it an essential step in object segmention.

The calculation formulas for mAP and AP are shown in Equations 3, 4, where p represents precision, r represents recall, and N represents the total number of sample categories. Since this study is a binary classification problem, the mAP value is the actual value of AP.


(3) $$\begin{array}{l} \int_{0}^{1}p\left(r\right)dr \end{array}$$

(4) $$mAP=\frac{\sum AP}{N}$$

## 4 Interpretation of result

**Figure 7:**
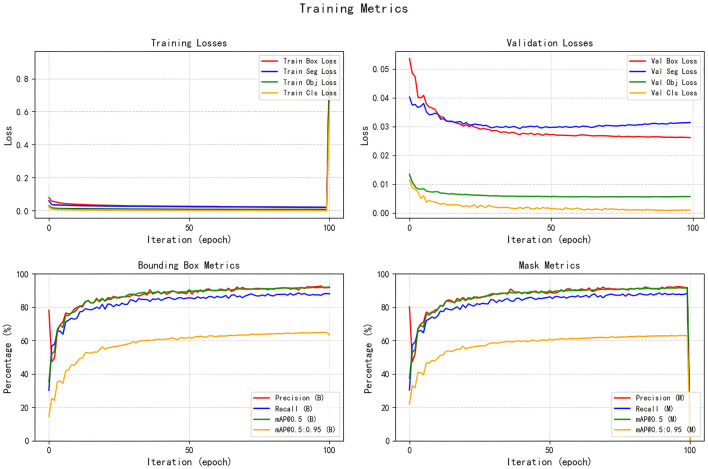
Training curve of the YOLOv5s-ASPP.

**Figure 8:**
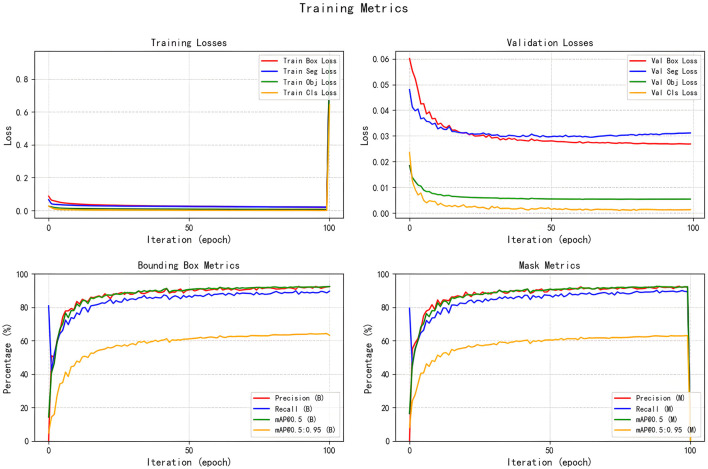
Training curve of the YOLOv5s-LAAM.

All seven models were trained for five rounds of 100 epochs each, with their average performance metrics on the validation set (Precision, Recall, mAP@50, and GFLOPs) summarized in Table 2, which compares the improved models' performance in brain tumor segmentation at IoU = 0.6. The experimental results indicate that YOLOv5s-ASPP and YOLOv5s-LAAM both exhibit significant performance improvements. The model with the LAAM module achieves the highest recall rate of 90.4 %, while maintaining a high mAP of 92.5 % and a lower computational load of 30.8 GFLOPs, reflecting a better efficiency balance. The APCM module proposed in this study shows accuracy and mAP@50 values comparable to YOLOv5s-ASPP, but its computational load is significantly higher than LAAM and ASPP. Overall, the LAAM module demonstrates superior performance. The training curves for YOLOv5s-ASPP and YOLOv5s-LAAM are shown in Figures 7, 8, respectively.

**Table 2:** Comparison of average values of evaluation metrics in ablation experiments.

**Name of model**	**Precision**	**Recall**	**mAP@50**	**GFLOPs**
YOLOv5	0.928	0.868	0.917	25.7
YOLOv5s-ASPP	0.937	0.891	0.929	32.3
YOLOv5s-LASPP	0.925	0.873	0.912	26.7
YOLOv5s-APCM	0.925	0.882	0.923	34.9
YOLOv5s-LAAM	0.923	0.904	0.925	30.8
YOLOv5s-LAPCM	0.912	0.882	0.917	26.7
YOLOv5s-LLAAM	0.914	0.873	0.913	26.5

To evaluate the performance advantages of our proposed YOLOv5s-LAAM model, we compared it with other recent YOLO-based brain tumor segmentation studies (32). As shown in Table 3, Ahsan et al. used a cascaded YOLOv5 and 2D U-Net model for pixel-level tumor segmentation on the Brain Tumor Figshare dataset, achieving a best mAP@50 of 89.5 % for detection. On the BRATS 2018 dataset, this model outperformed Mask R-CNN (mAP@50: 67 %).

**Table 3:** Performance comparison with existing brain tumor segmentation model studies.

**Name of model**	**mAP@50**
YOLOv5s-LAAM	0.925
YOLOv5-2D U-Net	0.895
Mask R-CNN	0.67

In contrast, our YOLOv5s-LAAM model achieved 92.3 % precision, 90.4 % recall, and an mAP@50 score of 0.925 on the internal test set. Despite dataset and task differences, our model showed higher mAP@50 within a single detection and segmentation framework. This confirms the LAAM module's effectiveness in enhancing feature extraction and segmentation. Also, our model maintained lower computational complexity with significantly reduced GFLOPs compared to the baseline model, highlighting its superior balance between accuracy and efficiency.

The study further evaluated the performance of the optimally trained weight files at IoU thresholds of 0.7, 0.8, and 0.9, with comparative results shown in Tables 4–6.

**Table 4:** Values of model evaluation indicators under IoU = 0.7.

**Name of model**	**Precision**	**Recall**	**mAP@50**
YOLOv5	0.924	0.88	0.917
YOLOv5s-ASPP	0.916	0.879	0.913
YOLOv5s-LASPP	0.920	0.873	0.903
YOLOv5s-APCM	0.919	0.88	0.916
YOLOv5s-LAAM	0.924	0.891	0.925
YOLOv5s-LAPCM	0.909	0.882	0.913
YOLOv5s-LLAAM	0.912	0.874	0.908

**Table 5:** Values of model evaluation indicators under IoU = 0.8.

**Name of model**	**Precision**	**Recall**	**mAP@50**
YOLOv5	0.918	0.874	0.908
YOLOv5s-ASPP	0.911	0.864	0.902
YOLOv5s-LASPP	0.920	0.859	0.899
YOLOv5s-APCM	0.924	0.869	0.906
YOLOv5s-LAAM	0.923	0.888	0.912
YOLOv5s-LAPCM	0.906	0.872	0.902
YOLOv5s-LLAAM	0.912	0.857	0.898

**Table 6:** Values of model evaluation indicators under IoU = 0.9.

**Name of model**	**Precision**	**Recall**	**mAP@50**
YOLOv5	0.894	0.837	0.881
YOLOv5s-ASPP	0.894	0.828	0.875
YOLOv5s-LASPP	0.896	0.827	0.871
YOLOv5s-APCM	0.902	0.838	0.879
YOLOv5s-LAAM	0.908	0.846	0.888
YOLOv5s-LAPCM	0.892	0.827	0.875
YOLOv5s-LLAAM	0.887	0.833	0.873

The evaluation of model performance under different IoU thresholds (0.7, 0.8, and 0.9) revealed that the YOLOv5s-LAAM model consistently demonstrated superior or leading performance in both recall and mAP@50. Particularly under the stringent condition of IoU = 0.9, it maintained the highest recall (84.6 %) and mAP@50 (88.8 %). In comparison, the baseline model and its ASPP variant exhibited more significant performance degradation. For instance, the ASPP version experienced a 4.2 % decline in mAP@50, whereas the LAAM version showed a more moderate decrease of only 3.7 %, highlighting its enhanced localization robustness.

Given the superior robustness and stability of the LAAM module at high IoU thresholds, the subsequent discussion will focus on comparing the performance differences between the ASPP and LAAM modules.

**Figure 9:**
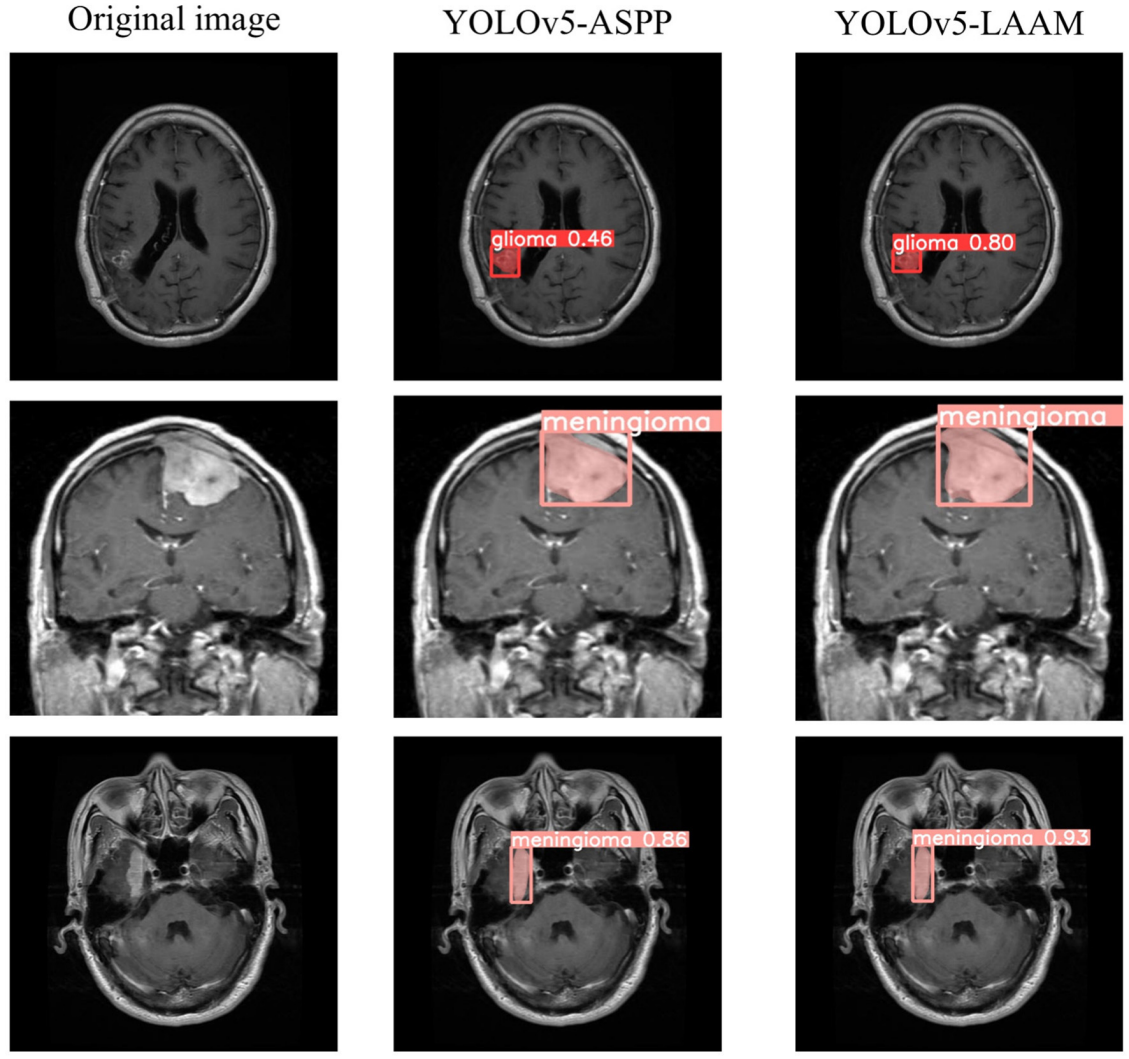
Test results of YOLOv5-ASPP and YOLOv5-LAAM on the internal test set.

**Figure 10:**
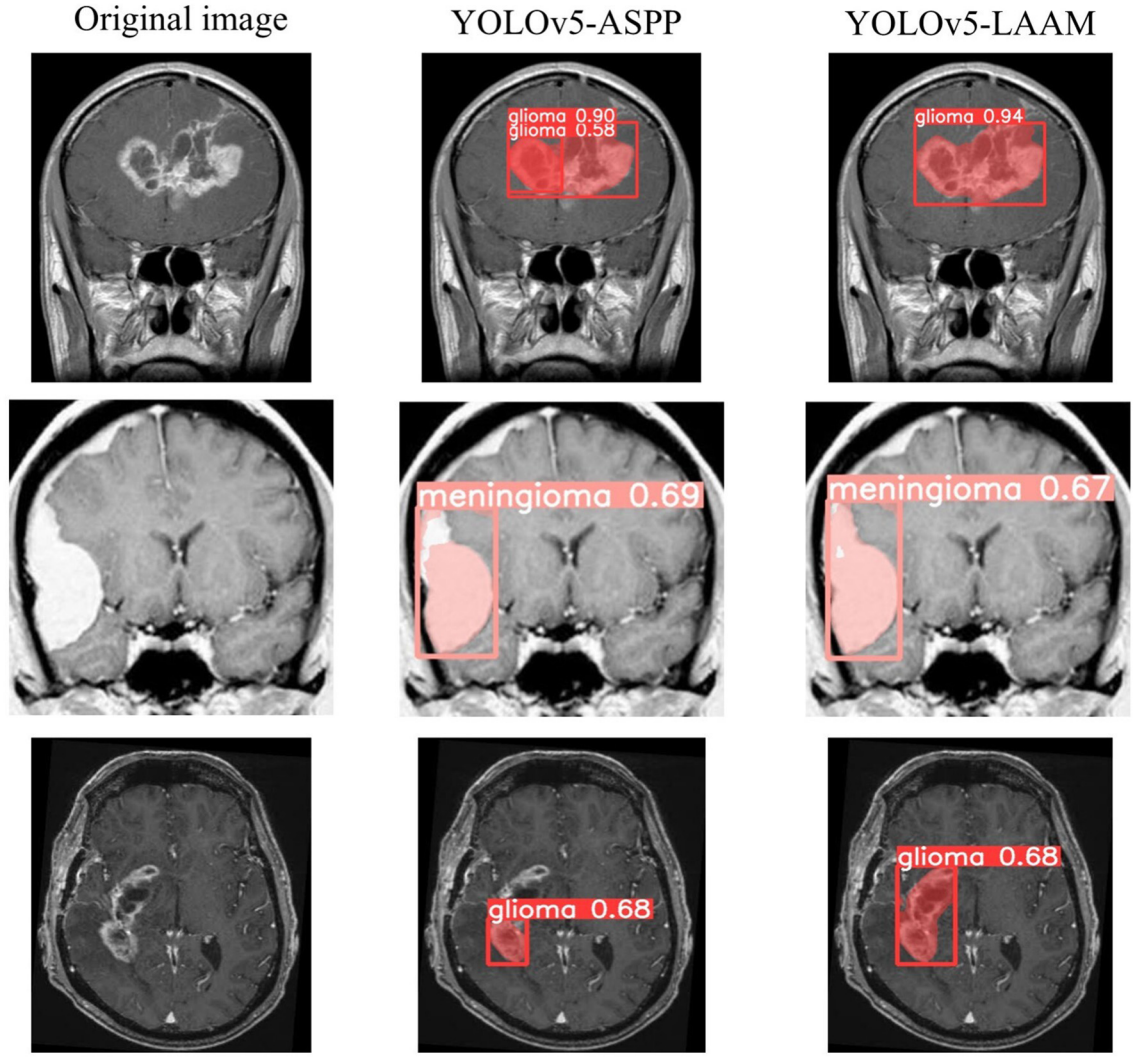
Test results of YOLOv5-ASPP and YOLOv5-LAAM on the external test set.

The training curves and optimal weight files of the YOLOv5s-ASPP and YOLOv5s-LAAM models, along with their performance on the internal and external test sets, are illustrated in Figures 7–10, and detailed in Tables 7, 8. Tables 9, 10 present the inference speed of the two models on the internal and external test sets.

**Table 7:** Test results on the internal test set of the model.

**Name of model**	***P-*value**	***R***	**mAP@50**
YOLOv5s-LAAM	0.842	0.824	0.833
YOLOv5s-ASPP	0.854	0.784	0.826

**Table 8:** Test results on the external test set of the model.

**Name of model**	***P-*value**	***R***	**mAP@50**
YOLOv5s-LAAM	0.748	0.741	0.751
YOLOv5s-ASPP	0.82	0.725	0.773

**Table 9:** Inference speed results for the internal test set of the model.

**Name of model**	**Pre-process Time (ms)**	**Inference Time (ms)**	**NMS Time (ms)**
YOLOv5s	0.4	8.5	1.3
YOLOv5s-LAAM	0.5	9.3	1.1
YOLOv5s-ASPP	0.5	9.6	1.4

**Table 10:** Inference speed results for the external test set of the model.

**Name of model**	**Pre-process time (ms)**	**Inference time (ms)**	**NMS time (ms)**
YOLOv5s	0.4	8.9	1.3
YOLOv5s-LAAM	0.4	9.7	1.1
YOLOv5s-ASPP	0.4	10.4	1.2

**Figure 11:**
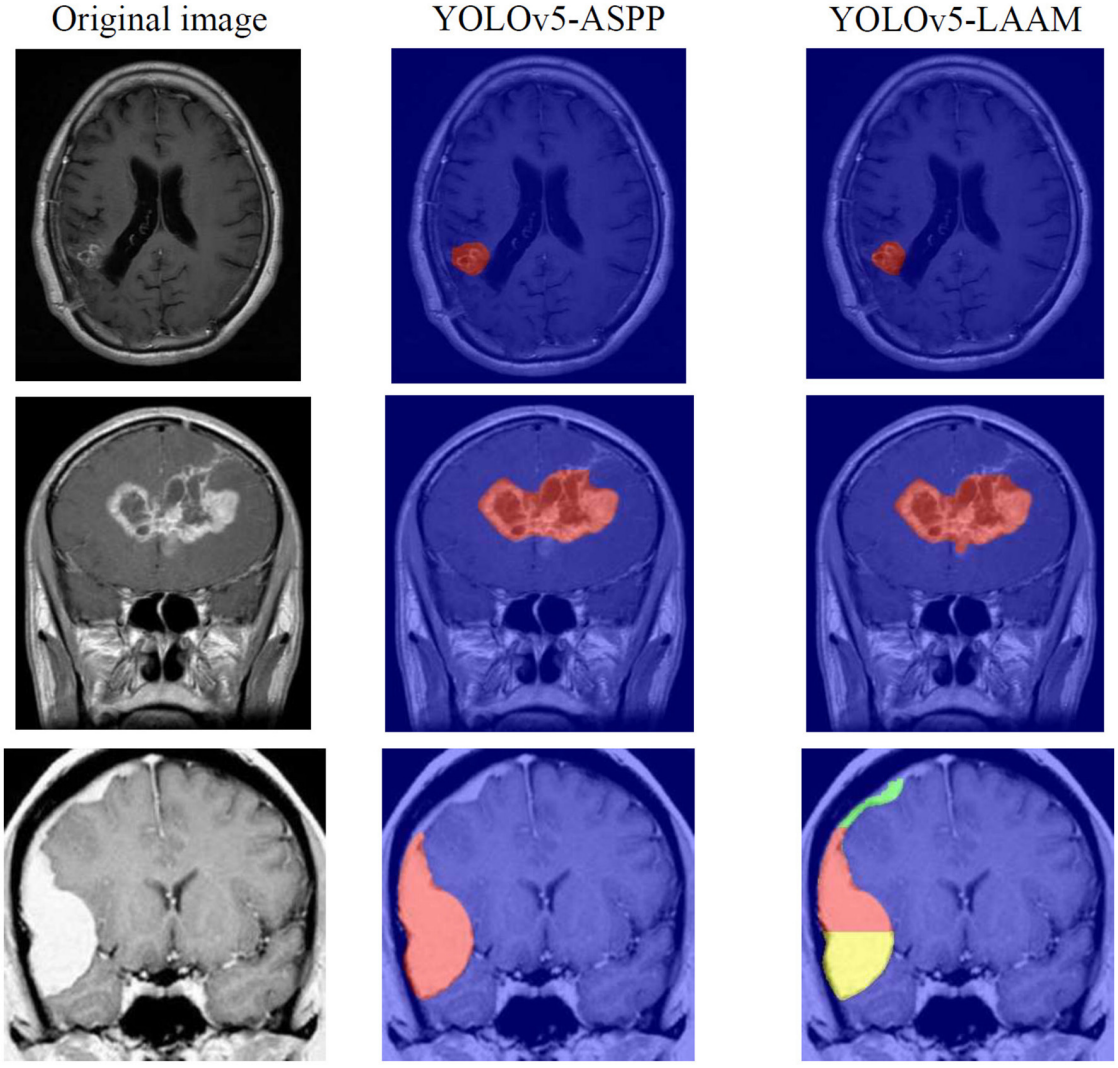
YOLOv5-ASPP and YOLOv5-LAAM visualization of heat maps through Grad-CAM.

The inference speed of the two models on the internal and external test sets is presented in Tables 9, 10. Grad-CAM was also used to visualize the heat maps of the YOLOv5-ASPP and YOLOv5-LAAM models, and the results are shown in Figure 11.

As shown in Tables 7, 8, on the internal test set, YOLOv5s-LAAM outperformed YOLOv5s-ASPP in both overall performance (mAP@50: 0.833) and recall rate (0.824), compared to the latter's scores (0.826 and 0.784, respectively). This indicates its superior lesion coverage capability and improved control over missed detections. Although its precision (0.748) on the external test set was lower than that of the ASPP variant (0.82), LAAM still maintained a higher recall rate (0.741). Furthermore, as illustrated in Figures 9, 10, it provided more complete and accurate localization and delineation of tumor regions.

As shown in Tables 9, 10, the YOLOv5s-LAAM model maintains high inference efficiency on both the internal and external test sets. Its inference time lies between that of the baseline YOLOv5s and YOLOv5s-ASPP models, indicating the LAAM module adds limited computational overhead despite incorporating multi-scale perception and attention mechanisms. Notably, the LAAM model's NMS Time is a consistent 1.1 ms in tests, showing its predictions are more concise with fewer redundant boxes. This highlights the model's good balance between accuracy and efficiency.

Grad-CAM visualization of the YOLOv5s-ASPP and YOLOv5s-LAAM models reveals marked differences in their attention distribution. As shown in Figure 11, the YOLOv5s-LAAM model generates more focused heat maps that closely match the actual tumor margins, with less activation of surrounding normal tissues. This reflects its superior lesion localization. In contrast, the YOLOv5s-ASPP model's heat maps are more dispersed, with some high-weight areas extending into normal tissues and incomplete coverage of the lesion, especially at the boundaries. Moreover, the YOLOv5s-LAAM model shows a multi-layer heat map color distribution within the lesion, indicating it can differentially respond to regions of varying significance. This confirms its strong feature perception and structural understanding. Overall, the visualization results demonstrate the YOLOv5s-LAAM model's superior lesion-focusing ability, consistent with its improved performance in quantitative metrics.

## 5 Discussion

In this study, a Lightweight Atrous Attention Module (LAAM) is proposed. By integrating the improved Atrous Spatial Pyramid Pooling (ASPP) multi-scale feature extraction and the Convolutional Block Attention Mechanism (CBAM), the performance of the YOLOv5s model in brain tumor MRI image segmentation is optimized. The Lightweight Atrous Attention Module (LAAM) employs depthwise separable convolution to reduce computational complexity and enhances key features dynamically through simplified channel-spatial attention, while improving training stability with residual connections.

The results show that the Lightweight Atrous Attention Module (LAAM) significantly outperforms the traditional Atrous Spatial Pyramid Pooling (ASPP) module in terms of recall rate (90.4 %) and mAP@50 (92.5 %), with a 15 % reduction in computational load (30.8 GFLOPs), demonstrating its ability to balance accuracy and efficiency. These results indicate that the Lightweight Atrous Attention Module (LAAM) can significantly improve the segmentation capability of the model, with high accuracy and potential for clinical implementation.

In our previous study, the YOLOv5s model integrated with the Atrous Spatial Pyramid Pooling (ASPP) also achieved good segmentation accuracy in the brain tumor segmentation task. This is because Atrous Spatial Pyramid Pooling enhances the feature representation capability through multi-scale dilated convolutions, resulting in high precision and mAP@50. However, it has a high computational complexity, limited ability to capture local details, and a fixed dilation rate design that cannot adapt to different target sizes, restricting its flexibility and efficiency in complex scenarios.

In contrast, the core advantage of the Lightweight Atrous Attention Module (LAAM) proposed in this study lies in its local attention mechanism's dynamic focusing ability on target regions. This mechanism significantly enhances the stability of recall by increasing sensitivity to key features. From IoU = 0.7 to 0.9, the recall rate of Lightweight Atrous Attention Module decreases by only 4.5 %, which is significantly lower than that of the baseline model (4.7 %) and Atrous Spatial Pyramid Pooling (ASPP) (5.1 %). This characteristic is particularly important in medical image analysis tasks.

The experimental data shows that the LAAM outperforms other models in recall rate across three IoU thresholds. This offers more complete tumor region localization for segmentation algorithms, enhancing the precision and continuity of segmentation boundaries. In clinical terms, the accuracy of brain tumor MRI image segmentation directly affects diagnostic precision and treatment plan making. The YOLOv5s-LAAM model proposed in this study significantly improves recall while maintaining high precision. This means the model can more comprehensively identify tumor regions, particularly in detecting small lesions or tumors with blurred boundaries. A high recall rate reduces the risk of missed diagnosis and provides clinicians with more reliable diagnostic support. Moreover, the model's lightweight design allows deployment in resource constrained medical settings, promoting the application of artificial intelligence in brain tumor diagnosis.

Moreover, during our other experiments and ablation studies, it was observed that although the Atrous Pyramid Convolution with Attention Module (APCM) significantly enhanced the model's adaptability to complex scenarios by integrating the multi-scale feature extraction of Atrous Spatial Pyramid Pooling (ASPP) with the attention mechanism of Convolutional Block Attention Mechanism (CBAM), its reliance on standard convolutions and a serial attention structure led to high computational complexity and limited inference efficiency, making it difficult to meet real-time requirements. Lightweight improved models, such as Lightweight Atrous Pyramid Convolution with Multi-scale Attention (LAPCM) and Lightweight Local-Attention Atrous Module (LLAAM), although having computational efficiency close to that of the baseline model, showed limited performance improvement, with some metrics even falling below those of the original model, indicating that further structural optimization is still needed. Overall, the Lightweight Atrous Attention Module (LAAM) achieves a better balance between accuracy, recall rate, and robustness, making it particularly suitable for high-precision medical tasks that are highly sensitive to missed detections.

The LAAM module demonstrates a remarkable performance in the internal testing, achieving an mAP@50 of 0.833 and a recall rate of 0.824. Nevertheless, in the external test set, there is a notable decline in precision (*P* = 0.748) compared to ASPP (*P* = 0.82), and the mAP@50 (0.751) is inferior to that of ASPP (0.773). This indicates that the generalization capability of the LAAM module has certain limitations.

The limitations of the LAAM module in cross-domain performance mainly result from its dynamic attention mechanism being deeply coupled with the statistical characteristics of the training data.

The images in dataset 1 have uniform quality, consistent contrast and noise patterns, providing a stable learning environment for the model. However, Compared with Dataset 1, Dataset 2 has differences in overall contrast and distinct noise textures due to the different acquisition devices, resulting in significant domain shift and posing challenges for model generalization.

During training, it learns attention mapping rules based on Dataset 1, which has consistent imaging quality, contrast distribution, and noise patterns. This enables LAAM to effectively capture typical tumor features and suppress specific background noise. However, when handling external test datasets, significant domain shifts in low-level statistical features occur due to differences in image acquisition devices, such as overall contrast changes and distinct noise textures. These shifts lead to mismatches in pre-trained attention weights. For instance, if tumor contrast generally decreases in the target domain, the pre-trained fully connected layer in the channel attention module calculates overly low weights due to input feature strength deviations, suppressing critical feature channels. Meanwhile, new noise patterns may be misclassified as significant spatial features by the spatial attention mechanism, causing false alarms.

Furthermore, the depthwise separable convolution in LAAM amplifies generalization deficiencies. While it operates efficiently on Dataset 1 due to consistent contrast distribution and noise patterns, its shared spatial convolution kernel mechanism significantly weakens cross-channel feature integration capabilities when facing differences like overall contrast discrepancies and unknown noise textures in Dataset 2. Subsequent 1 × 1 convolutional layers struggle to effectively compensate for these representation differences across domains. In contrast, the ASPP module employs a static multi-scale atrous convolution structure with a fixed receptive field design, focusing on extracting spatial context patterns rather than relying on data statistical characteristics. Thus, even with contrast differences or noise distribution changes in Dataset 2, ASPP can stably capture multi-scale spatial structural relationships, demonstrating stronger tolerance to low-level statistical shifts and superior cross-domain robustness (33). Future work could enhance LAAM's cross-domain generalization by incorporating adaptive fusion techniques.

In addition, although both the internal and external test sets contain meningiomas and gliomas, individual patient differences and the phenotypic diversity within the disease category, such as the heterogeneity of high and low-grade gliomas (LGG), remain an important factor affecting the cross-domain performance of the model.

The heterogeneity between high-grade gliomas (HGG) and low-grade gliomas (LGG) may also contribute to performance variation across datasets. HGGs are typically characterized by irregular morphology, infiltrative growth, and the presence of necrosis or edema, which leads to highly complex and diverse imaging patterns. In contrast, LGGs usually exhibit more homogeneous structures and relatively clear boundaries, yet their lower contrast against normal brain tissue makes delineation challenging. Moreover, differences in the prevalence of HGG and LGG cases can introduce distributional imbalance, while the uncertainty of manual annotations—caused by infiltrative margins in HGG or ambiguous borders in LGG—further increases noise in training. Collectively, these factors may lead to under-segmentation in LGG and unstable boundary prediction in HGG, thereby reducing the model's robustness and generalizability across external cohorts.

However, due to the lack of detailed patient clinical metadata, such as age, gender, tumor grade and molecular classification, in the publicly used datasets, we are unable to further validate the generalization ability of the model at the population statistics and disease subtype subdivision levels. The future research direction could involve collaborating with multi-center hospitals to obtain datasets containing rich clinical information for more thorough subgroup analysis, thereby more comprehensively verifying and enhancing the clinical practical value of the model.

To compare the relative performance of this study with that of previous works, we conducted a cross-study comparison with the U-Net-based segmentation method. Zhang et al. (34) proposed AGResU-Net, which achieved Dice coefficients of 0.876, 0.772, and 0.720 for whole tumor, core tumor, and enhanced tumor segmentation on the BraTS 2017 dataset, representing a strong performance benchmark in this field.

Although differences exist in the datasets, task settings, and evaluation criteria, the reported high mAP@50 (0.925), high recall rate (90.4 %), and high precision rate (92.3 %) demonstrate the model's exceptional capability in identifying and localizing tumor regions. Notably, these outcomes are achieved in a single, efficient framework, with the model sustaining millisecond-level inference speed and demonstrating high segmentation accuracy even at an IoU threshold of 0.9.

Therefore, this study and models such as AGResU-Net constitute methodological complementarity: AGResU-Net established a good performance benchmark in segmentation accuracy, while YOLOv5s-LAAM maintained competitive accuracy while significantly improving computational efficiency, providing a feasible alternative for clinical environments with real-time requirements. The above comparison results confirm that the advantages of the proposed model extend beyond performance optimization within the YOLO architecture and are demonstrated in medical image analysis tasks, showing good application potential and promotion value.

Overall, the YOLOv5s-LAAM proposed in this paper demonstrates advantages in both accuracy and efficiency. EoFormer (35), based on the Transformer architecture, effectively extracts tumor boundary features, but has a high computational complexity; the MFI method is good at handling multimodal missing issues (36), but has not fully optimized the computational efficiency and global feature extraction capability. The method proposed in this study achieves higher accuracy and recall rate while maintaining a lower computational cost, achieving a good balance between multi-scale feature perception, modality adaptability, and computational efficiency, and is more suitable for clinical practical environments.

This study has several limitations. First, the experiments were conducted only within the YOLOv5s framework. Future work will explore LAAM's generalization in Transformer frameworks for multimodal medical imaging. Second, the data sources were limited, which may insufficiently represent the diversity of clinical scenarios and affect the model's generalization. Third, despite improved computational efficiency, the model's real-time performance in medical settings, especially with high-resolution images, needs further optimization and testing.

Deep learning has broad applications in brain functional imaging. The STAD model innovatively combines diffusion models with a Transformer architecture, enabling high-quality spatial super-resolution reconstruction of EEG signals and enhancing classification and source localization (37). The PALH model integrates prior-guided adversarial learning and hypergraph structures to predict abnormal brain connections in Alzheimer's disease, offering strong neuroscientific interpretability (38). These models highlight the potential of generative models and multimodal fusion technologies in brain signal enhancement, brain network modeling, and disease prediction, and are expected to aid in building more accurate and interpretable clinical diagnostic frameworks for neurological disorders.

## 6 Conclusion

This study proposes a Lightweight Atrous Attention Module (LAAM) and applies it to the YOLOv5s model for segmenting brain tumor MRI images. The experimental results demonstrate that the improved YOLOv5s-LAAM model performs remarkably well in terms of precision, recall, and required computational resources, with a precision of 92.3 %, recall rate of 90.4 %, mAP@50 of 0.925, and a significant reduction in computational load, decreasing GFLOPs by 15 % compared to the original YOLOv5s-ASPP model. By integrating multi-scale dilated convolutions with an improved attention mechanism, the Lightweight Atrous Attention Module effectively enhances the model's segmentation capability for brain tumor MRI images, making it a powerful tool for clinical diagnosis and treatment planning.

## Data Availability

Publicly available datasets were analyzed in this study. This data can be found at: https://www.kaggle.com/datasets/masoudnickparvar/brain-tumor-mri-dataset; https://www.kaggle.com/datasets/sartajbhuvaji/brain-tumor-classification-mri.
