# Supplementary material for: Application research on YOLOv5 model based on Lightweight Atrous Attention Module in brain tumor MRI image segmentation
Source: Front Med (Lausanne). 2025 Oct 9;12:1660445. doi: 10.3389/fmed.2025.1660445 (PMC12546333; doi:10.3389/fmed.2025.1660445)
Supplement: Supplementary file 1 [file Table_1.docx]

| **Algorithm: Atrous Pyramid and CBAM Module (APCM)** **Input:** tensor x (B, Cin, H, W) **Output:** tensor out (B, Cout, H, W)   1. Multi-scale feature extraction (ASPP): f1 ← 1×1 Conv + ReLU(x) f2 ← 3×3 Conv (d=1) + ReLU(x) f3 ← 3×3 Conv (d=6) + ReLU(x) f4 ← 3×3 Conv (d=12) + ReLU(x) f5 ← 3×3 Conv (d=18) + ReLU(x) f6 ← GlobalAvgPool(x) → 1×1 Conv + ReLU → Upsample(H, W) aspp_out ← Concat(f1, f2, f3, f4, f5, f6) → BN → 1×1 Conv 2. Attention mechanism (CBAM): ca_out ← ChannelAttention(aspp_out) sa_out ← SpatialAttention(ca_out) 3. Feature fusion: fused ← Concat(aspp_out, sa_out) out ← 1×1 Conv(fused) |
| --- |

Algorithm A1. Pseudocode of the Lightweight Atrous Attention Module

| **Algorithm: Lightweight Atrous Attention Module (LAAM)**  **Input**: tensor x (B, Cin, H, W)  **Output**: tensor out (B, Cout, H, W)  1. Multi-scale feature extraction:  f1 ← 1×1 Conv + BN + ReLU(x)  f2 ← 3×3 Conv (d=6) + BN + ReLU(x)  f3 ← 3×3 Conv (d=12) + BN + ReLU(x)  aspp_out ← Concat(f1, f2, f3) → 1×1 Conv + BN + ReLU  2. Attention mechanism:  ch_att ← ChannelAttention(aspp_out)  sp_att ← SpatialAttention(aspp_out)  att_out ← aspp_out × ch_att × sp_att  3. Residual connection:  res ← 1×1 Conv + BN(x)  out ← att_out + res |
| --- |

Algorithm A2. Pseudocode of the Lightweight Atrous Attention Module
